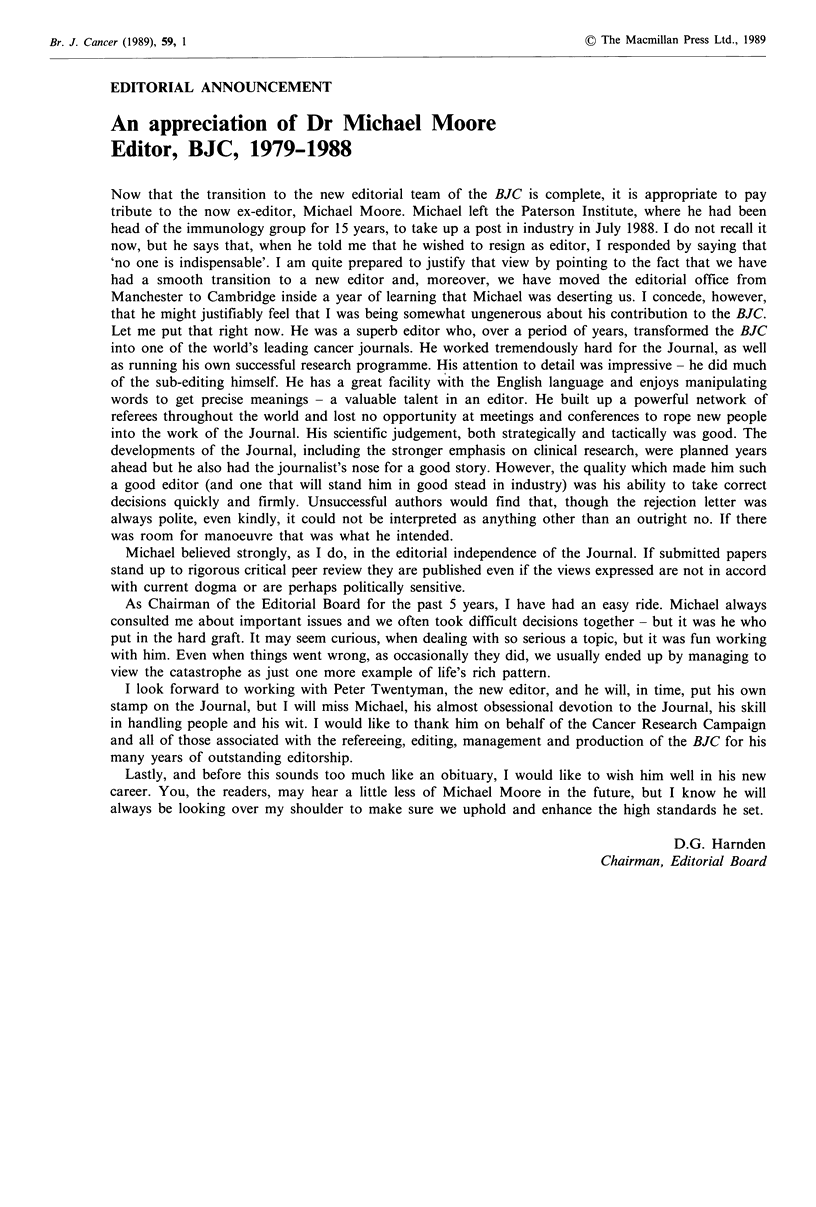# An appreciation of Dr Michael Moore, Editor, BJC, 1979-1988

**Published:** 1989-01

**Authors:** D.G. Harnden


*Br. J. Cancer* (1989), **59**, 1 © The Macmillan Press Ltd., 1989

EDITORIAL ANNOUNCEMENT

### An appreciation of Dr Michael Moore Editor, BJC, 1979–1988

Now that the transition to the new editorial team of the *BJC* is complete, it is appropriate to pay tribute to the now ex-editor, Michael Moore. Michael left the Paterson Institute, where he had been head of the immunology group for 15 years, to take up a post in industry in July 1988. I do not recall it now, but he says that, when he told me that he wished to resign as editor, I responded by saying that 'no one is indispensable'. I am quite prepared to justify that view by pointing to the fact that we have had a smooth transition to a new editor and, moreover, we have moved the editorial office from Manchester to Cambridge inside a year of learning that Michael was deserting us. I concede, however, that he might justifiably feel that I was being somewhat ungenerous about his contribution to the *BJC*. Let me put that right now. He was a superb editor who, over a period of years, transformed the *BJC* into one of the world's leading cancer journals. He worked tremendously hard for the Journal, as well as running his own successful research programme. His attention to detail was impressive – he did much of the sub-editing himself. He has a great facility with the English language and enjoys manipulating words to get precise meanings – a valuable talent in an editor. He built up a powerful network of referees throughout the world and lost no opportunity at meetings and conferences to rope new people into the work of the Journal. His scientific judgement, both strategically and tactically was good. The developments of the Journal, including the stronger emphasis on clinical research, were planned years ahead but he also had the journalist's nose for a good story. However, the quality which made him such a good editor (and one that will stand him in good stead in industry) was his ability to take correct decisions quickly and firmly. Unsuccessful authors would find that, though the rejection letter was always polite, even kindly, it could not be interpreted as anything other than an outright no. If there was room for manoeuvre that was what he intended.

Michael believed strongly, as I do, in the editorial independence of the Journal. If submitted papers stand up to rigorous critical peer review they are published even if the views expressed are not in accord with current dogma or are perhaps politically sensitive.

As Chairman of the Editorial Board for the past 5 years, I have had an easy ride. Michael always consulted me about important issues and we often took difficult decisions together – but it was he who put in the hard graft. It may seem curious, when dealing with so serious a topic, but it was fun working with him. Even when things went wrong, as occasionally they did, we usually ended up by managing to view the catastrophe as just one more example of life's rich pattern.

I look forward to working with Peter Twentyman, the new editor, and he will, in time, put his own stamp on the Journal, but I will miss Michael, his almost obsessional devotion to the Journal, his skill in handling people and his wit. I would like to thank him on behalf of the Cancer Research Campaign and all of those associated with the refereeing, editing, management and production of the *BJC* for his many years of outstanding editorship.

Lastly, and before this sounds too much like an obituary, I would like to wish him well in his new career. You, the readers, may hear a little less of Michael Moore in the future, but I know he will always be looking over my shoulder to make sure we uphold and enhance the high standards he set.

D.G. Harnden
*Chairman, Editorial Board*